# Insight into muscle stem cell regeneration and mechanobiology

**DOI:** 10.1186/s13287-023-03363-y

**Published:** 2023-05-12

**Authors:** Kuin Tian Pang, Larry Sai Weng Loo, Sean Chia, Francesca Yi Teng Ong, Hanry Yu, Ian Walsh

**Affiliations:** 1 Bioprocessing Technology Institute, Agency for Science, Technology and Research, Singapore, Singapore; 2 School of Chemistry, Chemical Engineering, and Biotechnology, Nanyang Technology University, 62 Nanyang Drive, N1.2-B3, Singapore, 637459 Singapore; 3 Institute of Bioengineering and Bioimaging, Agency for Science, Technology and Research, Singapore, Singapore; 4 Department of Physiology, Yong Loo Lin School of Medicine, National University of Singapore, Singapore, Singapore; 5 Mechanobiology Institute, National University of Singapore, Singapore, Singapore; 6 CAMP, Singapore-MIT Alliance for Research and Technology, Singapore, Singapore; 7 Interdisplinary Science and Engineering Program, NUS Graduate School, National University of Singapore, Singapore, Singapore

**Keywords:** Skeletal muscle stem cells, Mechanobiology, Stiffness, Viscoelasticity, Strain, Topography

## Abstract

Stem cells possess the unique ability to differentiate into specialized cell types. These specialized cell types can be used for regenerative medicine purposes such as cell therapy. Myosatellite cells, also known as skeletal muscle stem cells (MuSCs), play important roles in the growth, repair, and regeneration of skeletal muscle tissues. However, despite its therapeutic potential, the successful differentiation, proliferation, and expansion processes of MuSCs remain a significant challenge due to a variety of factors. For example, the growth and differentiation of MuSCs can be greatly influenced by actively replicating the MuSCs microenvironment (known as the niche) using mechanical forces. However, the molecular role of mechanobiology in MuSC growth, proliferation, and differentiation for regenerative medicine is still poorly understood. In this present review, we comprehensively summarize, compare, and critically analyze how different mechanical cues shape stem cell growth, proliferation, differentiation, and their potential role in disease development (Fig. 1). The insights developed from the mechanobiology of stem cells will also contribute to how these applications can be used for regenerative purposes using MuSCs.

## Introduction of muscle stem sells

### Functional role of muscle stem cells

**Fig. 1:**
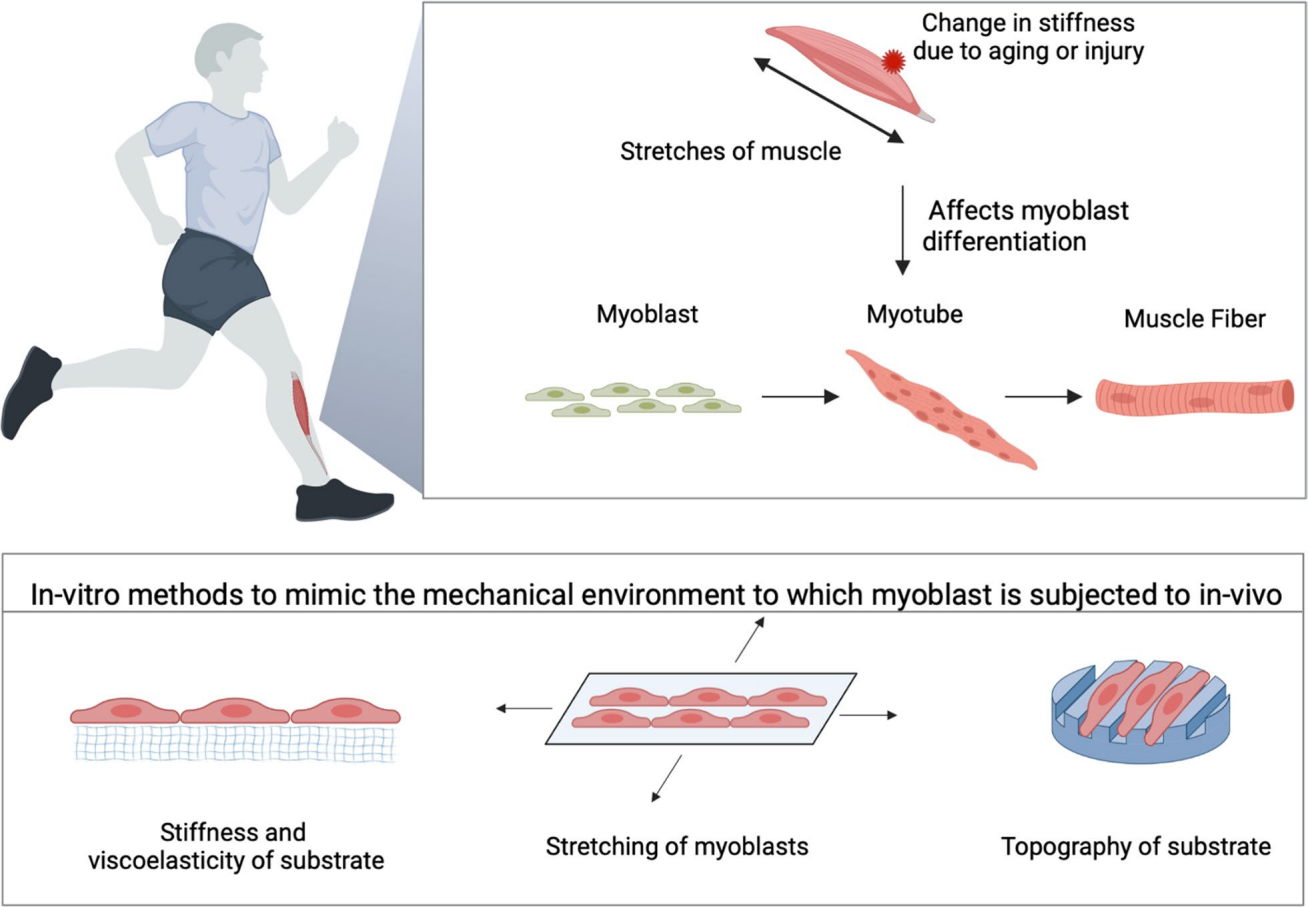
A schematic diagram that summarizes the role of mechanical forces in myoblast differentiation

Functional skeletal muscles are integral in daily activities, and they undergo consistent loading stress. When they are unable to support the excessive tensile strength, muscle injury inevitably occurs on post-mitotic cells that do not possess the capability to undergo cell division. As such, the main role of muscle tissue regeneration falls solely on MuSCs which play important roles in the growth, repair, and regeneration of skeletal muscle homeostasis [1]. They exist within quiescent cells in healthy adult mammals, accounting for around 2.5–6.5% of all muscle fiber-associated nuclei. In the resting state, they are mitotically quiescent in the G0 phase, suggesting the cells do not undergo active proliferation. These quiescent MuSCs are characterized by the expression of paired box transcription factor paired box 7 (Pax7) that plays an important role in the maintenance and self-renewal of MuSCs [2, 3] (Fig. 1).

The proliferative capability of MuSCs serves two purposes; (1) replenishment of the MuSCs pool in the stem cell niche and (2) generation of committed myoblasts that are key to muscle tissue regeneration. PAX7 is an important transcription factor that plays a role in skeletal precursor cells proliferation [3, 4], while myoblast determination protein 1 (MyoD) is one of the earliest key markers in muscle cell commitment and differentiation and increase in MyoD expression is critical for further differentiation of myoblasts into myotubes [5, 6]. To replenish the MuSCs niche, an individual MuSC undergoes symmetric cellular division to give rise to identical MyoD^low^/Pax7^high^ daughter cells that are capable of self-renewal to meet future demands of muscle tissue regeneration [7, 8]. When muscle tissue injury occurs, a small population of MuSCs undergoes asymmetric cellular division, with a self-renewing daughter clone (MyoD^low^/Pax7^high^) and a committed daughter clone (MyoD^high^/Pax7^low^) that exits the cell cycle. These MyoD^high^/Pax7^low^ committed cells eventually differentiate into myoblasts expressing MyoD, myogenic regulatory factor 4 (Mrf4) and myogenic regulatory factor 5 (Myf5) [9]. Subsequent differentiation of these myoblasts into myofibers is accompanied by the decrease of Pax3/7 and Myf5 expression and an increase of myogenin and myosin heavy chain (MHC) [10, 11]. As such, muscle tissue homeostasis is balanced by the constant self-renewal process of (MyoD^low^/Pax7^high^) MuSCs that are capable of self-renewal and MyoD^high^/Pax7^low^ MuSCs for further commitment toward repairing muscle tissue damage. In fact, when Pax7 + MuSCs are eliminated in mice through transgenic targeting of Pax7^Cre^, the mice experienced > 90% failure in muscle regeneration [12–15].

Inside the skeletal muscle, MuSCs reside in a specialized niche that is located between the muscle fiber plasma membrane and the basal lamina that surrounds the muscle fiber [16–18]. Inside this specialized niche, the muscle fiber basal lamina consists of extracellular matrix (ECM) components such as entactin, fibronectin, laminin and type IV collagen [19] and MuSCs are able to interact with them through the expression of a7- and B1-integrins [20]. As a result, various growth factors such as basic fibroblast growth factors (bFGF), epidermal growth factor (EGF) and hepatocyte growth factor (HGF), and insulin-like growth factor-1 (IGF-1) promote the survival and proliferation of MuSCs [21–24].

### Muscle stem cells in aging and their influence on cell microenvironment

MuSCs possess a high regenerative potential which can be used to replenish lost MuSCs. However, the regenerative capacity of the MuSCs declines during the natural aging process; the properties of muscle tissues start to change as the muscle stem niche starts to undergo remodeling [25]. Damage to skeletal muscle tissue eventually results in fibrosis and stiffening of the extracellular matrix (ECM) [26, 27]. Consequently, the microenvironment of MuSCs is remodeled, suggesting that a greater collagen deposition increases muscle stiffness [28]. This stiffness of the microenvironment may be due to alterations to collagen and advanced glycation end-products (AGE), which impair MuSCs proliferation [29]. Many studies have shown that the aging MuSC niche also plays a key role in the impairment of self-renewal ability in MuSCs [30–33].

There is still controversy over the aging effects that result in the loss of MuSCs. While some studies have revealed that there were no differences in MuSC counts in young and aged muscles [34–36], others have shown that estimated MuSC counts increase or decrease with age [37]. Aged MuSCs exhibit impaired proliferative potential due to a delayed response to activation [34, 38]. These aged MuSCs also exhibit susceptibility to tumor necrosis factor-α (TNF-α)-induced apoptosis [39, 40]. Rejuvenation of activation ability in aging muscle stem cells is dependent on physical exercise that restores Cyclin D1 expression.

## Current challenges in the differentiation and proliferation processes of muscle stem cells

MuSCs are a rare population of cells, accounting for around 2.5–0.5% of all muscle fiber-associated nuclei. Due to the limited amount found in the human body, several groups have started to look at other types of stem cells such as mesenchymal stem cells (MSCs). There are several pieces of evidence showing the potential of MSCs giving rise to MuSCs after transplantation into mdx mouse model [41, 42]. Stem cells are undifferentiated cells that possess the ability to undergo self-renewal and differentiate into different lineages such as cardiac [43], hepatic [44], neuronal [45], and pancreatic [46, 47]. In general, stem cells can be differentiated in a stepwise guided process using growth factors, chemicals, and small molecules of which developmental potency decreases at each specific stage. The differentiated endpoint cells can be used for cell and drug therapies.

The development and growth of skeletal muscle cells from the embryo stage to the adult stage are generally well understood. For the past few decades, researchers have also been searching for ways to generate skeletal muscle cells from pluripotent stem cells in vitro. These efforts are made possible with our understanding of the biochemical and molecular aspects of directed differentiation and directed reprogramming of stem cells at different stages to skeletal muscle cells [48]. Traditionally, the activation of signaling pathways was often carried out by using specific growth factors, small molecules, and chemicals. However, usage of these growth factors is very costly due to the short half-life of the proteins. Moreover, these growth factors may inevitably induce certain unintended signaling pathways that may be detrimental to the survival, proliferation, and differentiation of MuSCs.

While MuSCs have been shown to promote muscle regeneration, they often exhibit loss of potency when they are expanded ex vivo. MuSCs also exhibit limited in vivo migration, suggesting limited regenerative potential in injury sites. It has also been shown that MuSCs rapidly lose their stemness with the removal of their stem cell niche [49, 50]. There is an urgent need to recapitulate the MuSC niche in vitro so that they are able to remain in a quiescent state. A cocktail combination of 4 different factors, interleukin 1α (IL-1α), interleukin 13 (IL-13), tumor necrosis factor-α (TNF-α) and interferon γ (INF-γ), promoted the proliferation and differentiation of myosatellite cells for more than 20 passages. The myosatellite cells that are expanded ex vivo under such conditions expressed high *Pax7* and low *MyoD* expression levels and were able to repopulate the stem cell niche [51].

Mechanical forces are being investigated to understand how they can complement the use of growth factors in promoting MuSC regeneration and maintaining the stemness of muscle stem cells. In addition, in consideration of the fact that skeletal muscle tissues are always under mechanical loading and stretching, it makes sense to investigate the role of mechanical forces in promoting MuSCs proliferation and differentiation, which in turn results in improved muscle regeneration. In the following section, we review such influences.

## Mechanobiology of skeletal muscle stem cells

In the body’s natural microenvironment, stem cell fate and proliferation are constantly regulated by biophysical cues. Biological cells are known to sense mechanical forces and their mechanical environment and translate these mechanical cues into biochemical signals. Examples include the effect of fluidic shear stress, strain, and ECM stiffness on endothelial cells [52, 53], smooth muscle cells [54], and cancer cells [55], respectively. Similarly, cells of the musculoskeletal system are also capable of sensing mechanical forces. Additionally, it has been observed that skeletal muscle cells change their size and structural properties in response to mechanical stimulation [56].

Stem cells are also capable of sensing mechanical cues and mediating their differentiation accordingly. They sense mechanical forces and the environment through various mechanosensory mechanisms. Cell density, shape, and the adhesion area of human mesenchymal stem cells (hMSC) have been shown to drive their commitment switch between osteogenic or adipogenic fates—larger adhesion area drives hMSC commitment toward osteogenesis and activates ras homology family member A (RhoA) expression. RhoA then signals through its effector—Rho-associated protein kinase (ROCK) to activate myosin II and cytoskeletal tension activity [57]. Another signaling pathway that is involved in the mechanosensing of hMSC is the Yes-associated protein/Taffazin (YAP/TAZ) pathway. The spreading of hMSC on a larger adhesion area increases the localization of YAP/TAZ in the nucleus and this regulation is independent of the Hippo pathway. In addition, ECM stiffness, which is known to regulate hMSC, also affects YAP/TAZ activation. Regulation of stiffness-mediated differentiation of hMSC is abrogated when YAP/TAZ pathway is inhibited, suggesting the important role of YAP/TAZ pathway in the mechanoregulation of hMSC differentiation. YAP/TAZ activity also requires the tension of the actin cytoskeleton and Rho [58]. Cell spread is measured by cell aspect ratio—a ratio between width and length; the ratio was found to influence stem cell lineage commitment in a microfilament and ROCK-dependent manner [59]. The aforementioned evidence shows that stem cells sense their mechanical environment partially through adhesion and cytoskeletal activity. These will be discussed in detail in the next section.

## Influence of mechanical factors on muscle stem cell properties

### Substrate stiffness

Stiffness, commonly known as rigidity, is a mechanical property that measures the resistance of the material to deformation. It is measured by Young’s modulus—a ratio between stress and strain (*E* = *σ*/*ε*) in the unit of Pascals. Stiffness characterization of biological cells and ECM have been shown to be important for understanding cellular physiology, development, and pathological progression of diseases [60–65]. In particular, ECM stiffness has been shown to regulate cell migration [66, 67], apoptosis [68], and proliferation [69]. ECM stiffness is also known to govern stem cell behavior. For example, adipose-derived stem cells cultured on hydrogels with a linear stiffness gradient revealed that cell aspect ratio and expression of mechanosensitive markers lamin A, nucleus YAP translocation, and myocardin-related transcription factor A (MRTFa) were positively correlated to substrate stiffness via myosin and Rho/ROCK signaling [70].

Out of all mechanical cues, ECM stiffness plays a major role in stem cell differentiation. hMSC commits to lineages toward the cell type where in vivo stiffness matches the stiffness of the matrix. For instance, while soft polyacrylamide gels that mimic brain and fat tissue environment drive neurogenic and adipogenic differentiation respectively, stiff polyacrylamide gels that mimic the bone drive osteogenic differentiation [58, 71]. Cytoskeleton tension is involved in stiffness-regulated differentiation. A similar observation was also made when MSCs were cultured within 3-dimensional arginylglycylaspartic acid (RGD)-alginate microspheres [72]. Hence, stem cells would differentiate into skeletal muscle cells if they were grown on ECM with stiffness that emulates those of skeletal muscles.

To maintain MuSCs stemness, a 3D micro scaffold (~ 1–2 kPa) composed of collagen, recombinant laminin and α4β1 integrin was used in muscle stem cell culture and they were able to maintain muscle cell stemness [73]. The stiffness of mouse skeletal muscle and myoblast cell lines (C2C12) was found to be around 12 kPa through atomic force microscopy (AFM) indentation [74, 75]. Myoblasts were also found to exhibit a higher level of differentiation, striation, and fusion to myotubes when they were cultured on collagen-coated polyacrylamide gel substrates that mimic the stiffness of skeletal muscles (~ 12 kPa). Interestingly, there was little striation when myoblasts were cultured on stiff glass substrates [75].

Human bone marrow- and dental-derived MSCs, or gingival MSCs, were able to undergo myogenic differentiation when they were encapsulated in alginate microspheres (enriched with myogenic cocktail) with well-tuned stiffness. Both MSC grown on alginate microspheres with a stiffness of 15 kPa, compared with 5 and 45 kPa, had the highest expression of MyoD protein, suggesting that a stiffness of 15 kPa (stiffness close to the stiffness of skeletal muscle) provides an optimal mechanical environment for myogenic differentiation [76].

Self-renewal of stem cells is essential because they replicate and regenerate more stem cells to help in tissue regeneration. MuSCs cultured on soft poly(ethylene glycol) (PEG) hydrogel substrates that have a stiffness of 12 kPa, compared with cells cultured on stiff plastic dishes, exhibited self-renewal phenotype in vitro*.* These stem cells were successfully transplanted into mice and contributed to muscle regeneration [77]. It has also been shown that hematopoietic and progenitor cells cultured on elastic tropoelastin had a higher expansion of undifferentiated cells compared with culture multiwell plates [78]. Human MuSCs were also shown to remain quiescent and maintain their ‘stemness’ when they were cultured on soft poly-L-lysine/hyaluronic acid films, while their counterparts cultured on stiff films were activated and began to differentiate and proliferate [79].

Changes in stiffness can occur in muscle injury. Muscle stiffness was found to increase from around 13–20 kPa after mice muscles were injured using barium chloride (BaCl_2_), and this stiffness remained elevated after completion of muscle regeneration 28 days after injury. Further, strain-promoted azide–alkyne cycloaddition (SPAAC) hydrogels with a PEG precursor were used to mimic the stiffness of injured muscles and myoblast cell line (C2C12) cells cultured on them exhibited increased proliferation and migration, and localization of YAP/TAZ to the cell nucleus [80]. A similar observation was also made by increasing muscle stiffness from 12 kPa to approximately 25 kPa in the first three days post-injury [81]. Importantly, PEG-based hydrogel stiffness was found to synergize with NOTCH signaling to regulate muscle stem cell fate—Jagged-1 tethered on stiff hydrogel (42 kPa) as compared with soft hydrogel (12 kPa), reduced myoblast myogenin expression and that is dependent on ROCK [81]. Isolated shrunken and collapsed myofibers cultured ex vivo were also found to have significantly higher stiffness (2 kPa) compared with their intact counterparts (0.5 kPa) and the provision of a stiffer microenvironment for satellite cells in turn promoted muscle progenitor cells (MPCs) proliferation. MPCs that were grown on a 2 kPa polyacrylamide-based substrate that mimics collapsed myofibers stiffness exhibited higher proliferation and reduced spontaneous differentiation compared to MPCs grown on a 0.5 kPa [82]. At the cellular level, MuSCs stiffness increased by 2.9-fold during skeletal muscle regeneration after BaCl_2_-induced injury. High 3-dimensional niche stiffness (21.7 kPa) increased the amount of planar orientation of self-renewal division via the Wnt family member 7a (Wnt7a) and non-canonical Wnt pathway, as compared with low 3D niche stiffness (5.9 kPa) [62].

ECM stiffness has also been studied in the context of skeletal muscle aging, a condition that is characterized by declining muscle strength, which leads to reduced mobility and muscle function. This condition has been partly attributed to the defects in MuSC regeneration [25]. Aged muscles that were injured were found to have high ECM deposition, rather than undergoing myofiber repair (Grounds, 1998), which in turn increases muscle stiffness [83, 84]. MuSCs that were grown on decellularized aged ECM (with high stiffness) exhibited lower myogenic and higher fibrogenic markers than those grown on decellularized young ECM. Fibroblasts grown on stiff silicone gels (32 kPa) that mimic the stiffness of aged muscle expressed higher YAP/TAZ nuclear translocation and secreted soluble mediators that inhibit myogenesis [84]. Furthermore, another study found that only one-third of MuSCs from aged mice retain their renewal ability and capacity to repair myofibers. This defect was rescued by culturing aged muscle stem cells on soft PEG-based hydrogel (12 kPa) in conjunction with p38α and p38β inhibition. Interestingly, tuning substrate stiffness alone was not sufficient to enhance cell renewal suggesting that mechanical and biochemical cues are required to synergistically modulate stem cell renewal in aged muscle [85].

### Substrate viscoelasticity

Most studies characterized the effect of ECM stiffness on stem cell fate. However, it is important to note that ECM-like fibrin and collagen are viscoelastic materials [86, 87]. Hence, investigating the effect of elasticity alone does not uncover the true mechanobiological behavior of stem cells. In contrast to pure elastic material, viscoelastic materials exhibit a time-dependent response to an applied load due to the flow and remodeling of the ECM. In particular, ECM exhibits time-dependent stress relaxation: cells cultured on a viscoelastic material first experience the stiffness of the material, and then the traction force of cells reduces as ECM is relaxed over time. Cell spreading and proliferation on viscoelastic materials that exhibit stress relaxation were larger than those cultured on purely elastic ECM with the same stiffness. Fibroblasts cultured on viscoelastic material were also found to have high nuclear translocation of YAP than those cultured on pure elastic materials. These effects are mediated through actomyosin-based contractility and integrin adhesions [88].

Consistent with the above observation [88], hMSCs cultured on polyacrylamide gel with higher loss modulus, a measure of energy dissipation under dynamic mechanical testing, exhibited higher spread area, greater proliferation, and differentiation potential. Interestingly, hMSCs were more sensitive to changes in loss modulus, as compared with the change in stiffness. Changing the loss modulus by two orders of magnitude resulted in the same fold-change in cell spread area as differences in stiffness by three orders of magnitude. In the presence of the inductive myogenic, adipogenic, and osteogenic medium, hMSCs cultured on the substrate with higher loss modulus expressed significantly higher levels of differentiation markers, suggesting that higher loss modulus of the substrate is more favorable for MSC differentiation. This observation was attributed to higher cell spread area and loss of cytoskeletal tension through the loss of energy resulting from material creep within the substrates with high loss modulus [89]. The same group went on to prove that substrate loss modulus-mediated Rac1 activation, increased N-Cadherin expression, and higher hMSCs motility and lamellipodial protrusion rates are the underlying mechanisms for enhanced differentiation toward smooth muscle cell lineage [90]. Another study by Chaudhuri et al. (2016) also supported this observation—MSCs spreading, proliferation, YAP nuclear translocation, and osteogenic differentiation were enhanced when they were cultured on polyacrylamide hydrogels with faster relaxation time [91].

Although ECM viscoelasticity has an important role in stem cell differentiation, its role in skeletal stem cell differentiation has not been investigated. This remains a research gap that should be filled to have a comprehensive understanding of the role of ECM mechanics on skeletal stem cell differentiation.

### Stretching

Skeletal muscle is a mechanically dynamic tissue, and they contract and relax up to 17% of their length to produce voluntary motion [92]. Mechanical strain has been shown to result in skeletal muscle strengthening and hypertrophy [93] and maintenance of muscle progenitors [94]. Hence it is important to understand the role of mechanical strain in the regeneration of skeletal muscle cells—particularly self-renewal and myogenesis of muscle stem cells and their precursor cells. In most studies, mechanical strain is characterized as a ratio of change in length (transverse or longitudinal length) to the original length; it is often expressed as a percentage.

Biaxial cyclic strain (10%, 0.17 Hz) was found to inhibit hESC differentiation, as evidenced by the upregulated expression of Oct4 and SSEA-4. Mechanical strain also promoted hESC self-renewal while maintaining their pluripotency. Medium conditioned by strained-hESCs did not inhibit unstrained-hESC differentiation, suggesting that only mechanical force, but not other soluble mediators, was responsible for the inhibition of differentiation [95]. The same group went on to demonstrate that the same mechanical strain-induced TGFβ1, activin A, Nodal expression and Smad 2/3 phosphorylation are responsible for strain-induced inhibition of hESC differentiation and stimulation of self-renewal [96]. However, mechanical strain does not inhibit commitment in all types of lineages. Cyclic strain (10%, 1 Hz) inhibited osteogenic and neurogenic differentiation of bone marrow-derived MSCs but promoted smooth muscle differentiation of these cells. Round cells, among other cell shapes, were most sensitive to mechanical strain, while spindle cells and amebocytes were only responsive to mechanical strain for vascular smooth muscle and neurogenic differentiation [97].

In the context of skeletal muscle regeneration, MSCs, myoblast, and skeletal MuSCs were stretched to investigate the effects of strain on stem cell proliferation and differentiation. Mouse myoblast (C2C12) subjected to uniaxial strain (12.2–14.2%, 0.5 Hz) aligned perpendicular to the direction of strain [98]. This has been shown to be dependent on strain amplitude as other studies that applied lower strain magnitude (7%, 0.5 Hz, and 2%, 1 Hz) reported lower orientation angle (7% strain) or no alignment (2% strain) [99, 100]. Alignment was not observed when C2C12 were subjected to equibiaxial strain (approximately 10%, 0.5 Hz). Moreover, equibiaxial strain resulted in cell membrane damage. Uniaxial strain, but not equibiaxial stain, enhanced myogenic differentiation, evidenced by myotube/myoblast ratio and myosin-positive myotubes [98]. In contrast to that, a study has shown that equibiaxial strain (3%, 0.05 Hz) also enhanced myotube formation [101]; the cause for the discrepancy observed between this study and Pennisi et al. (2011)’s is not clear.

Not all studies supported the effect of strain in the inhibition of differentiation: In a study by Boonen et al. (2010), C2C12 and MPCs subjected to uniaxial ramp strain (0–2%) for two days, followed by intermittent dynamic strain (2–6%, 3 h on, 3 h off, 1 Hz) showed suppressed myotube formation and maturation [100]. High uniaxial cyclin strain (17%, 1 Hz) was also found to promote the proliferation of C2C12 and inhibited myotube formation in C2C12 culture via the activation of NF-κΒ, Ras-related C3 botulinum toxin substrate 1 (Rac-1), and focal adhesion kinase (FAK) [102]. The inconsistency of the effect of uniaxial strain is likely due to the lower or higher strain magnitude (less than 10% or above 15%) and different strain application strategies adopted by them.

Skeletal muscle inflammation can result in muscle protein degradation and inhibit myogenesis [103]. Myotube formation was inhibited in C2C12 treated with pro-inflammatory cytokine TNF-α through the activation of NF-κB pathway and induction of nitric oxide synthase 2A (NOS2A) and nitric oxide (NO) production [104, 105]. Equibiaxial cyclic strain (3, 6, 9, 12, and 18%, 0.05 Hz) was found to inhibit TNF-α-induced expression of NOS2 in a magnitude-dependent manner, and it (3%, 0.05 Hz) also rescued impairment of myogenesis caused by TNF-α [101].

The effect of mechanical strain on muscle stem cells was also investigated. Primary isolated satellite cells, as opposed to transformed myoblastic cell lines such as C2C12, provide better insight into how mechanical strain affects myogenesis as these cells are stem-like cells that differentiate into myoblasts in vivo. Mechanical strain (10%, 0.25 Hz) promoted bovine satellite cell proliferation and reduced myogenic differentiation via the activation of extracellular signal-regulated kinase (ERK) [106].

Although skeletal muscle stem cells are the most studied cell type in tissue regeneration, its lack of differentiation capacity poses a challenge for use in therapeutics. Other sources of stem cells such as adipocyte-derived stem cells (ASCs) and bone marrow-derived mesenchymal stromal cells (BMSCs) are sought after as an alternative to skeletal muscle stem cells. ASCs can participate in muscle regeneration by fusion or co-culturing with myoblasts to form myotubes [107–109]. Uniaxial strain (15%, 0.5 Hz) has been shown to enhance ASCs fusion with skeletal myocytes. More myotubes were formed when ASCs are strained while cocultured with mouse skeletal muscle myoblasts (C2C12) [110]. As for BMSCs, uniaxial strain (10%, 0.17 Hz) enhanced myogenic differentiation and reduced proliferation and motility. Strain also aligned BMSCs parallel to the strain direction, and this is different from what was reported in myoblasts [98, 110, 111].

### Topography

Topographical cues can significantly affect the cellular morphology and phenotype of various cell types such as epithelial cells [112, 113], fibroblasts [114], and endothelial cells [115]. Stem cells were also found to be influenced by topography. ESCs grown on Polydimethylsiloxane (PDMS) gratings with 600 nm features and spacing showed stronger alignment and elongation. The same study also showed that nanotopographic cues altered the organization of various cytoskeletal components, including F-actin, vimentin, γ-tubulin, and α-tubulin, and the observed changes in proliferation and morphology were abolished by the effect of actin-disrupting agents like cytochalasin D and latrunculin B [116].

Skeletal muscles consist of highly aligned multinucleated myotubes and their alignment is required for myotube fusion [117]. During in vivo myogenesis, grooves formed between muscle fibers have a depth and width of between 1–4.5 μm and 2–3 μm, respectively. Myoblasts grew and aligned themselves along the grooves and differentiated into well-aligned and fused skeletal muscle tissue [118]. Therefore, it is not surprising that topography can direct stem cell alignment and differentiation toward myogenesis. Primary myoblasts and C2C12 myoblasts were found to align well along grooves with a range of subcellular widths between 2 and 75 μm [119–124]. In addition to width, the depth of grooves was found to affect myoblast alignment too. C2C12 cells seeded in 2 μm wide and 7 μm deep PDMS grooves aligned better than those seeded in 2 μm wide and 2 μm deep grooves—this is likely because the shallow grooves did not provide sufficient contact guidance for cell alignment. C2C12 cells seeded in 2 μm wide and 7 μm deep grooves also had better formation and fusion of multinucleated myotubes [124]. Grooves are also found naturally in cellulose. Decellularized green onion has natural grooves measuring 20 μm wide by 10 μm deep, and C2C12 myoblasts grown on them were aligned with more myotube formation [120]. Similar observations were also made when C2C12 myoblasts were grown on submicron grooves made of polystyrene, polycaprolactone, and PEG-based hydrogel [125–127].

With advances in biomaterials, the effect of nanoscale topography on muscle stem cells can be investigated. For example, cellulose nanowhiskers (CNWs) extracted from *Ascidiella aspersa* and *Halocynthia roretzi* have widths of 6–7 nm and 10–15 nm, respectively. C2C12 myoblasts seeded on this nanoscale topography showed a higher degree of multinucleated myotube fusion with fibrillar fibronectin deposition on the surface of highly oriented CNWs [128, 129]. Similarly, C2C12 cells grown on alginate nanofibers made using a modified electrospinning process had higher adhesion, alignment and myotube formation [127].

MSCs derived from bone marrow, fetal tissue, and adipose grown on microcontact printed-20 μm wide fibronectin lanes had aligned actin filaments and vinculin, and elongated nuclei. The alignment caused by the lanes was found to drive myogenic differentiation in these cells too [130]. Similarly, ASCs seeded in 100 μm wide and 27 μm deep grooves made of polyacrylamide hydrogel exhibited a higher level of alignment and myotube fusion compared to unpatterned control [131]. Some studies have also attempted to grow cells on a complex 3D contour. For instance, a study developed a novel molding technique to replicate human skeletal muscle myoblast features and contour by creating an inverted pattern on poly(urethane acrylate)(PUA). hMSCs grown on 3D ‘pockets’ with myoblasts features took the shape and alignment of the topography and are committed to myogenesis, compared with hMSCs grown on a flat control [132].

## Conclusion

In summary, MuSCs possess high regenerative capabilities for muscle tissue repair and regeneration. However, current understanding and knowledge of MuSCs biology were always based on 2D monolayer cultures on a tissue culture dish. This outdated approach does not recapitulate the in vivo 3D microenvironment where external mechanical cues like substrate stiffness, strain, and topography play important roles in MuSC survival and proliferation. Moreover, the biological effects whereby MuSCs are governed by mechanobiological forces are not well-studied. Hence, there is a need to carry out in vitro 3D studies to understand how mechanobiological forces govern MuSCs self-renewal, proliferation, and survival. Further work may involve high-throughput screening of MuSC response to mechanobiological cues to elucidate novel mechano-signaling pathways. Such approaches may support the use of mechanobiology forces to prime MuSCs for enhanced survival and proliferation. This will also lead to the discovery of certain drugs that will augment MuSCs growth. One possible suggestion would be to incorporate both aspects of biomechanical cues (novel biomaterials and external mechanical forces) and exogenous biological signals (growth factors such as EGF, HGF, and IGF) to promote muscle tissue regeneration. A thorough understanding of the mechanical forces regulating MuSCs allows for the development of bio-inspired extracellular matrix which can serve to increase mature muscle fiber differentiation.

Yet, our current understanding of how biomechanical cues and biochemical signals orchestrate is far from comprehensive. Extensive characterization of mechanobiological aspect of MuSCs renewal, survival, and differentiation needs to be carried out. The combination of the therapeutic effects of growth factors into novel biomaterials with well-tuned mechanics would help push the frontiers of skeletal muscle regeneration.

## Data Availability

Not applicable.

## Abbreviations

MuSCs: Muscle stem cells
Pax7: Paired box 7
MyoD: Myoblast determination protein 1
Mrf4: Myogenic regulatory factor 4
Mrf5: Myogenic regulatory factor 5
Pax3: Paired box 3
MHC: Myosin heavy chain
bFGF: Basic fibroblast growth factors
EGF: Epidermal growth factor
HGF: Hepatocyte growth factor
IGF-1: Insulin-like growth factor 1
ECM: Extracellular matrix
AGE: Advanced glycation end-product
TGFβ: Transforming growth factor β
NF-κB: Nuclear factor kappa B
IL-6: Interleukin 6
DMD: Duchenne muscular dystrophy
DGC: Dystrophin glycoprotein complex
IL-1α: Interleukin 1α
IL-13: Interleukin 13
TNF-α: Tumor necrosis factor-α
INF-γ: Interferon γ
hMSC: Human mesenchymal stem cells
RhoA: Ras homology family member A
ROCK: Rho-associated protein kinase
YAP: Yes-associated protein
TAZ: Taffazin
MRTFa: Myocardin-related transcription factor A
RGD: Arginylglycylaspartic acid
PEG: Poly(ethylene glycol)
SPAAC: Strain-promoted azide–alkyne cycloaddition
MPC: Muscle progenitor cells
Wnt7a: Wnt family member 7a
Rac-1: Ras-related C3 botulinum toxin substrate 1
FAK: Focal adhesion kinase
NOS2A: Nitric oxide synthase 2A
NO: Nitric oxide
ERK: Extracellular signal-regulated kinase
ASCs: Adipocyte-derived stem cells
BMSCs: Bone marrow-derived mesenchymal stromal cells
PDMS: Polydimethylsiloxane
CNWs: Cellulose nanowhiskers
PUA: Poly(urethane acrylate)
